# A simple label-free electrochemical sensor for sensitive detection of alpha-fetoprotein based on specific aptamer immobilized platinum nanoparticles/carboxylated-graphene oxide

**DOI:** 10.1038/s41598-021-93399-y

**Published:** 2021-07-07

**Authors:** Jantima Upan, Napaporn Youngvises, Adisorn Tuantranont, Chanpen Karuwan, Philippe Banet, Pierre-Henri Aubert, Jaroon Jakmunee

**Affiliations:** 1 Department of Chemistry, Faculty of Science, Chiang Mai University, Chiang Mai, 50200 Thailand; 2 Thammasat University Research Unit in Carbon Materials and Green Chemistry Innovations, Department of Chemistry, Faculty of Science and Technology, Thammasat University, Pathum Thani, 12120 Thailand; 3 Graphene and Printed Electronics for Dual-Use Applications Research Division, National Security and Dual-Use Technology Center, National Science and Technology Development Agency, Pathumthani, 12120 Thailand; 4 Center of Advanced Materials of Printed Electronics and Sensors, Materials Science Research Center, Faculty of Science, Chiang Mai University, Chiang Mai, 50200 Thailand; 5 CY Cergy Paris Université, LPPI, 95000 Cergy-Pontoise, France; 6 Center of Excellence for Innovation in Chemistry, and Center of Chemistry for Development of Health Promoting Products From Northern Resources, Faculty of Science, Chiang Mai University, Chiang Mai, 50200 Thailand

**Keywords:** Sensors, Nanoscale materials

## Abstract

A label-free electrochemical aptamer-based sensor has been fabricated for alpha-fetoprotein (AFP) detection. Platinum nanoparticles on carboxylated-graphene oxide (PtNPs/GO-COOH) modified screen-printed graphene-carbon paste electrode (SPGE) was utilized as an immobilization platform, and the AFP aptamer was employed as a bio-recognition element. The synthesized GO-COOH helps to increase the surface area and amounts of the immobilized aptamer. Subsequently, PtNPs are decorated on GO-COOH to enhance electrical conductivity and an oxidation current of the hydroquinone electrochemical probe. The aptamer selectively interacts with AFP, causing a decrease in the peak current of the hydroquinone because the binding biomolecules on the electrode surface hinder the electron transfer of the redox probe. Effects of aptamer concentration and AFP incubation time were studied, and the current changes of the redox probe before and after AFP binding were investigated by square wave voltammetry. The developed aptasensor provides a linear range from 3.0–30 ng mL^−1^ with a detection limit of 1.22 ng mL^−1^. Moreover, the aptamer immobilized electrode offers high selectivity to AFP molecules, good stability, and sensitive determination of AFP in human serum samples with high recoveries.

## Introduction

Hepatocellular carcinoma (HCC) is considered the most common primary cancer of the liver that causes people's deaths worldwide. Alpha-fetoprotein (AFP) is widely considered an important biomarker for HCC^1^, normally produced by the liver, yolk sac, and gastrointestinal tract of humans. Generally, the AFP level in serum of healthy humans should be lower than 25 ng mL^−1^, while the increment of AFP to 500 ng mL^−1^ could be suspected as HCC patients^2^. Therefore, the quantitative analysis of AFP concentration is essential for early clinical diagnosis and long-term treatment. Several publications have been reported for AFP detection, for example, enzyme-linked immunosorbent assay^3^, fluorescent immunosensor^4,5^, chemiluminescent immunosensor^6^, and electrochemical immunosensor^7–9^. Among these methods, an electrochemical immunosensor is attracted much attention due to its high sensitivity and easy to fabricate, especially a label-free electrochemical immunosensor. Its principle is based on the immuno-interaction between AFP antibody and AFP protein^10–13^ that causes a change in the sensor's current response.

Although antibody is usually used as a recognition biomolecule to bind with AFP antigen specifically, there are some limitations such as low stability, easy degradation, expensive, and using an animal for the production. Due to these problems, an aptamer, a short single-strand deoxyribonucleic acid or a ribonucleic acid molecule, has been extensively studied as a useful alternative recognition element instead of an antibody^14–16^. Advantages such as low cost, easy to large-scale synthesis, excellent reproducibility, and good stability are achieved. Yang et al*.*^17^ reported a label-free electrochemical aptasensor utilizing graphene oxide modified GCE as a working electrode (WE) for the AFP detection. They synthesized graphene oxide (GO) consisting of many carboxylic groups to immobilize with NH_2_-functionalized AFP aptamer covalently. Their fabricated aptasensor offers reasonable specificity and stability for one week (the response remained 96%). However, GCE is not suitable for constructing a disposable and portable electrochemical immunosensor because an external auxiliary electrode (AE) and a reference electrode (RE) are required to perform the electrochemical measurement. In addition, it is high cost, non-disposable, and necessary to polish the electrode surface before use.

Numerous combined screen-printed electrodes (SPEs) containing WE, AE, and RE, have been used in various electrochemical sensors, especially those employing carbon as WE due to their low cost, large surface area, high reproducibility, and ease of mass production^18–20^. Moreover, SPEs allow for experiments with a small volume of solution, as little as a few microliters that can miniaturize the overall size of the analysis system and allow for the development of a portable electrochemical sensor^21^. Modification of WE surface can also be easily performed to obtain better analytical performance. As a growing interest of nanomaterials, they were extremely used to modify the electrode as their intrinsic features, for example, inexpensive, good thermal stability, and high surface area. For a number of years, GO nanosheet is getting much attention in biological applications for biomolecule immobilization because it has a large specific surface area and abundant functional groups such as carboxylic groups that can effectively bind with amine groups of biorecognition elements^22,23^. Jumpathong et al*.*^24^ developed a label-free electrochemical immunosensor for detection of human immunoglobulin G. They exploited the oxygenated groups as an antibody-immobilizing platform. The anti-IgG antibodies were covalently immobilized on the electrode surface by using amide coupling. Furthermore, to improve the detection's sensitivity, an electrochemical reaction of the redox probe must be enhanced. Therefore, noble metal nanoparticles are interesting to use for signal amplification owing to their unique properties, including high surface area, excellent conductivity, and catalytic activity, that can increase the electron transfer rate of the electrode^25,26^.

In the present study, we developed a novel label-free electrochemical aptasensor to sensitively determine AFP using a highly specific aptamer and nanocomposites of platinum nanoparticles on carboxylated-graphene oxide (PtNPs/GO-COOH). The low-cost and portable screen-printed graphene-carbon paste electrodes (SPGEs) were fabricated and utilized as the sensing platform. Carboxylated-graphene oxide was synthesized to obtain plenty of carboxylic groups on the graphene oxide that would efficiently promote immobilizing aptamer molecules to improve the detection sensitivity. Furthermore, some metal nanoparticles such as gold, platinum, and palladium incorporated with carboxylated-graphene oxide were investigated to increase the current response of the hydroquinone that was used as a redox probe. Morphology and electrochemical response of the sensing electrode were investigated by scanning electron microscopy and cyclic voltammetry, respectively. Finally, the developed aptasensor performance and its application to serum sample analysis were studied.

## Experimental section

### Chemicals and apparatus

All chemical reagents were of analytical grade. Deionized water was obtained from a system of Milli–Q, Millipore, Sweden. Alpha-fetoprotein (AFP) was purchased from Fitzgerald, USA. Bovine serum albumin (BSA), chloroacetic acid, chloroplatinic acid hexahydrate, and human serum sample were from Sigma-Aldrich, USA. Hydroquinone, *N*-(3-dimethylaminopropyl)-N′-ethylcarbodiimide hydrochloride (EDC), and graphite powder were received from Sigma-Aldrich, China, United Kingdom, and Switzerland, respectively. Sodium hydroxide and sodium chloride were from Loba Chemie, India. Potassium chloride was purchased from Ajax Finechem, Australia, and potassium dihydrogen phosphate was from Carlo Erba, Australia. Sodium phosphate dibasic dihydrate and N-Hydroxysuccinimide (NHS) were received from Merck, Germany. The functionalized aptamer was received from Biolegio, Netherland, with the following sequence^27^ of 5′-NH_2_-GTGACGCTCCTAACGCTGACTCAGGTGCAGTTC-TCGACTCGGTCTTGATGTGGGTCCTGTCCGTCCGAACCAATC-3′.

All electrochemical experiments were carried out by using an Emstat potentiostat (PalmSens BV, Netherland). The combined SPGEs were obtained from National Security and Dual-Use Technology Center, National Science and Technology Development Agency, Pathumthani, Thailand. The electrochemical platform comprises three electrodes, including SPGEs as WE and AE, and silver/silver chloride (Ag/AgCl) as RE^28^ The morphology and size of the synthesized materials were characterized by a transmission electron microscope (TEM, JEM 2010, Jeol, Japan). The surface of different electrodes was revealed by a scanning electron microscope (SEM, JSM 6335 F, Jeol, Japan). A Fourier transform infrared (FTIR) spectrum of the carboxylated-graphene oxide (GO-COOH) was recorded on an FTIR spectrometer (Thermo Scientific, Massachusetts, USA).

### Preparation of GO-COOH

GO was synthesized from graphite powder using the modified Hummers method^29^. Then, it was further functionalized with carboxylic acid using the reported procedure^30^. Firstly, 400 mg of GO was suspended in 100 mL of DI water by sonication for 1 h. Next, 24 g of sodium hydroxide and 20 g of chloroacetic acid were added to the suspension, and the mixture was continuously sonicated for 3 h. The suspension was neutralized with dilute HCl and purified by repeatedly rinsing and centrifugation. After that, the GO-COOH was dialyzed (using the dialysis membrane, MWCO:1 kDa, Spectrum Laboratories, Inc., USA) against DI water for 48 h to remove any ions and impurities. Finally, the GO-COOH was washed with DI water several times and dried in an oven at 60 ºC.

### Synthesis of PtNPs/GO-COOH

The PtNPs decorated GO-COOH can be prepared as follow; a portion of 50 mg of GO-COOH was dispersed in 50 mL of DI water by sonication for 20 min. Afterward, chloroplatinic acid hexahydrate solution was added to obtain a 10% weight of platinum. After sonication for 30 min, 10 mL of NaBH_4_ solution (4 mg mL^-1^) was added dropwise, and the suspension was continuously stirred for 2 h. The mixture was neutralized by washing with DI water. The precipitate was then collected using centrifugation and dried in an oven at 80 ºC to obtain the PtNPs/GO-COOH.

### Fabrication of the aptasensor

**Figure 1:**
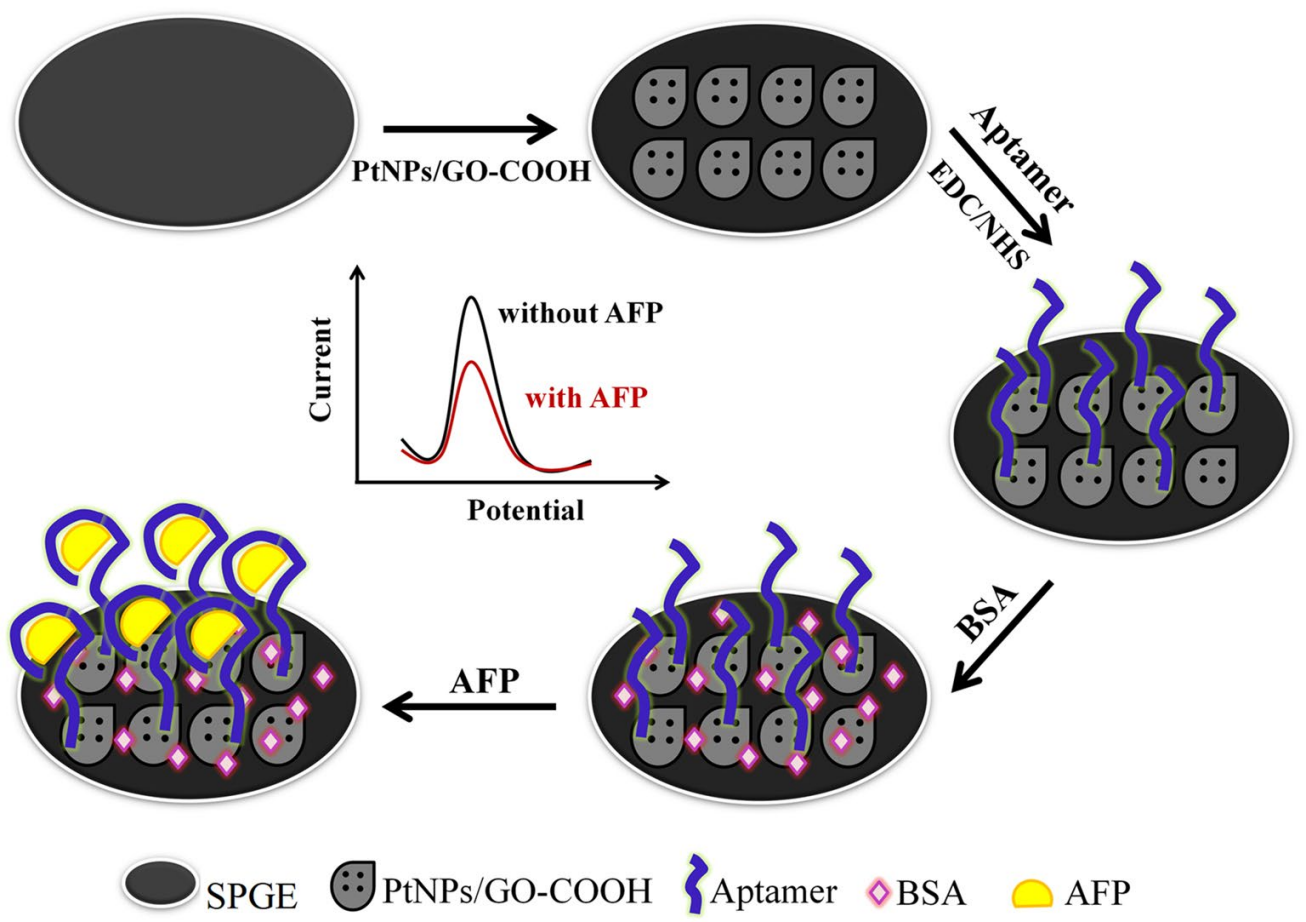
The stepwise preparation of the aptasensor.

The fabrication procedure of the aptasensor is shown in Fig. 1. Briefly, a graphene-carbon paste (WE) with 2.00 mm diameter was modified with 15 μL of 2 mg mL^−1^ of PtNPs/GO-COOH. The carboxylic groups were activated using 10 μL of the mixture of 0.4 M EDC and 0.1 M NHS (1:1v/v) solution for 30 min and washed with 10 mM PBS pH 7.4. Then, 5 µL of NH_2_-aptamer (7 µM, dissolved in PBS) was immobilized on the electrode for 45 min at room temperature. Next, the electrode (Apt/PtNPs/GO-COOH/SPGE) was washed with PBS to remove unbound aptamers. Subsequently, the electrode was incubated in BSA solution (0.25%w/v in PBS) at room temperature for 30 min to block the remaining active sites of the electrode surface and avoid the non-specific binding in the further step. After washing the electrode with PBS, the BSA/Apt/PtNPs/GO-COOH/SPGE was stored at 4 °C for later use.

### Electrochemical detection of AFP

To carry out the electrochemical detection of AFP, the initial peak current response, I_0_, before AFP incubation was recorded in 50 μL of 3 mM hydroquinone (prepared in PBS solution) by scanning a square wave voltammetric (SWV) potential from -1.0 to + 1.0 V. Then, 5 μL of different AFP concentration was dropped on the surface of the BSA/Apt/PtNPs/GO-COOH/SPGE to incubate for 45 min at room temperature. After the electrode was washed three times with PBS solution, a new SWV sweep was performed in 50 μL of 3 mM hydroquinone, and the peak current response, I_p_, was measured. The change of electrochemical response before and after AFP incubating step was expressed as the percentage of decreasing current.

$$Decreasing~current~\left( \% \right) = \left( {\frac{{I_{0} - I_{p} }}{{I_{0} }}} \right) \times 100$$

## Results and discussion

### Investigation of a working electrode

**Figure 2:**
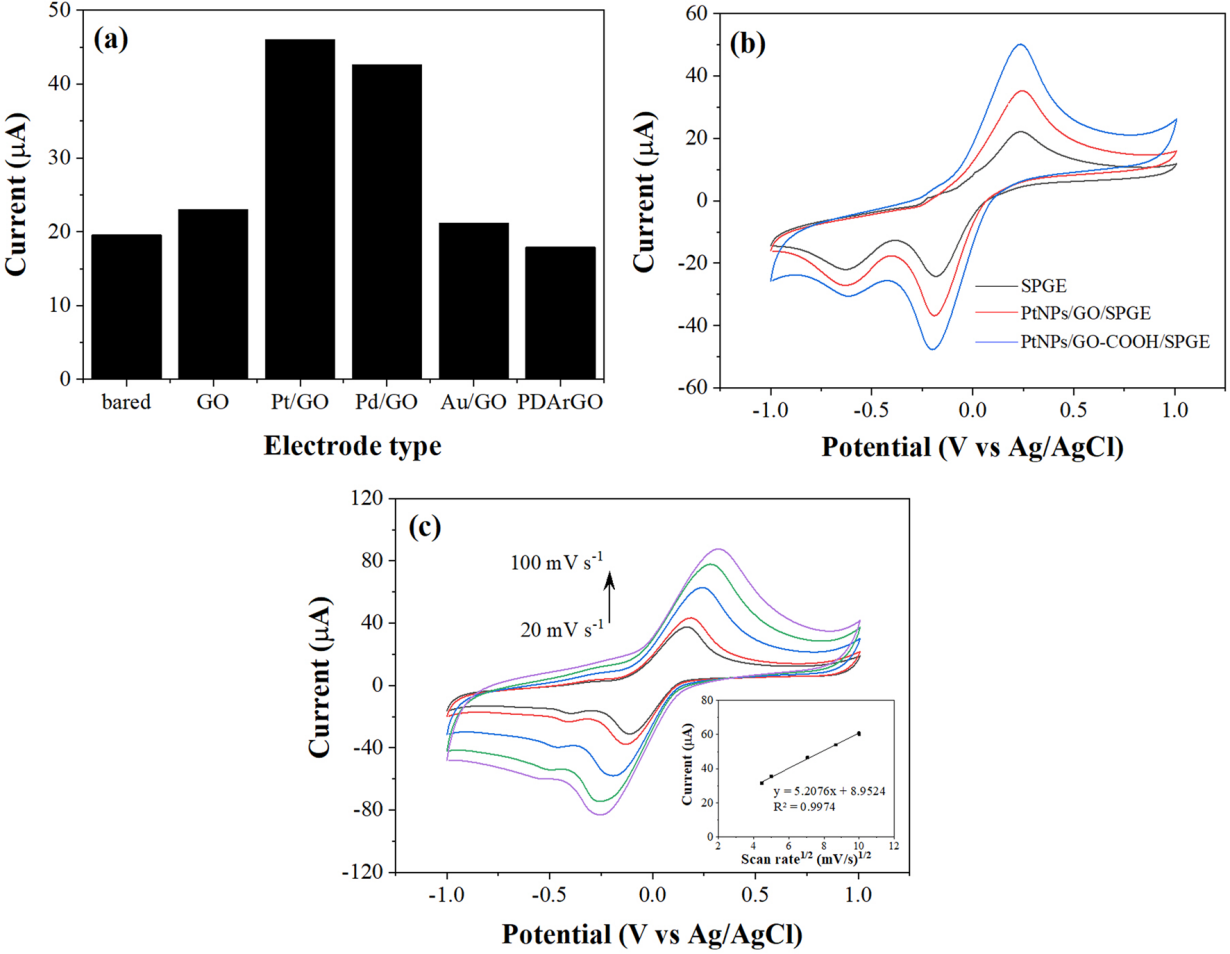
(**a**) Anodic current responses of various modified SPGEs, (**b**) CVs of SPGE, PtNPs/GO/SPGE, and PtNPs/GO-COOH/SPGE in 10 mM PBS solution pH 7.4 containing 3 mM hydroquinone at a scan rate of 100 mV s^−1^, and (**c**) CVs of hydroquinone at different scan rates.

We investigated suitable materials for the SPGE working electrode modification to obtain a high electrochemical probe's current response. In this work, hydroquinone was chosen as a redox probe because it is an excellent redox mediator. It provides high current responses both of oxidation and reduction reactions at low potential^31,32^, so it could be used as an alternative redox probe instead of ferri-ferro cyanide, hydrogen peroxide, and so on. The anodic peak currents of 3 mM hydroquinone on a bared SPGE and different modified SPGEs were investigated by CV in 3 mM hydroquinone as depicted in Fig. 2a. The result shows that an SPGE modified with PtNPs on GO obviously provided the highest anodic peak current. The anodic peak potential of hydroquinone on various electrodes are not apparently different (i.e., SPGE 0.239 V, GO 0.209 V, PtNPs/GO 0.229 V, and AuNPs/GO 0.119 V), indicating that the catalytic effect is not obviously seen. The large current signal enhancement obtained could be resulted from the high surface area and excellent electrical conductivity of the modified electrodes. Therefore, the PtNPs/GO/SPGE was selected as a WE for further use.

The carboxylic group is one of the best-known functional groups that can efficiently conjugate with biological molecules via covalent binding^30,33^. As mentioned in the introduction, GO can be functionalized interestingly with carboxylic groups to provide more reactive sites and further increase the immobilized aptamer molecules on the modified electrode. Next, electrochemical characteristics of a bared SPGE and SPGE modified with PtNPs/GO, and PtNPs/GO-COOH on the oxidation–reduction of hydroquinone were investigated as presented in Fig. 2b. In this figure, the oxidation peak at + 229 mV is the oxidation peak of hydroquinone, whereas the first reduction peak at − 191 mV is attributed to the quinone reduction. The second peak is observed about − 630 mV that belong to the reduction of oxygen^34^. The PtNPs/GO-COOH modified SPGE gave the highest current response to the redox probe. This could be explained by the higher hydrophilicity of GO-COOH offered by hydrophilic COOH groups. Besides, this can provide a good dispersion of Pt nanoparticles on dispersed GO-COOH sheets.

After that, the effect of the scan rate of the redox reaction on the modified electrode was studied from 20 to 100 mV s^−1^. It was found that the anodic peak current (I_pa_) of hydroquinone increased with increment of scan rate as shown in Fig. 2c. The I_pa_ is linearly proportional to the square root of scan rate with R^2^ of 0.9974 indicating that hydroquinone's redox process at the PtNPs/GO-COOH/SPGE is a diffusion-controlled reaction.

### Characterization of PtNPs/GO-COOH and the modified electrodes

**Figure 3:**
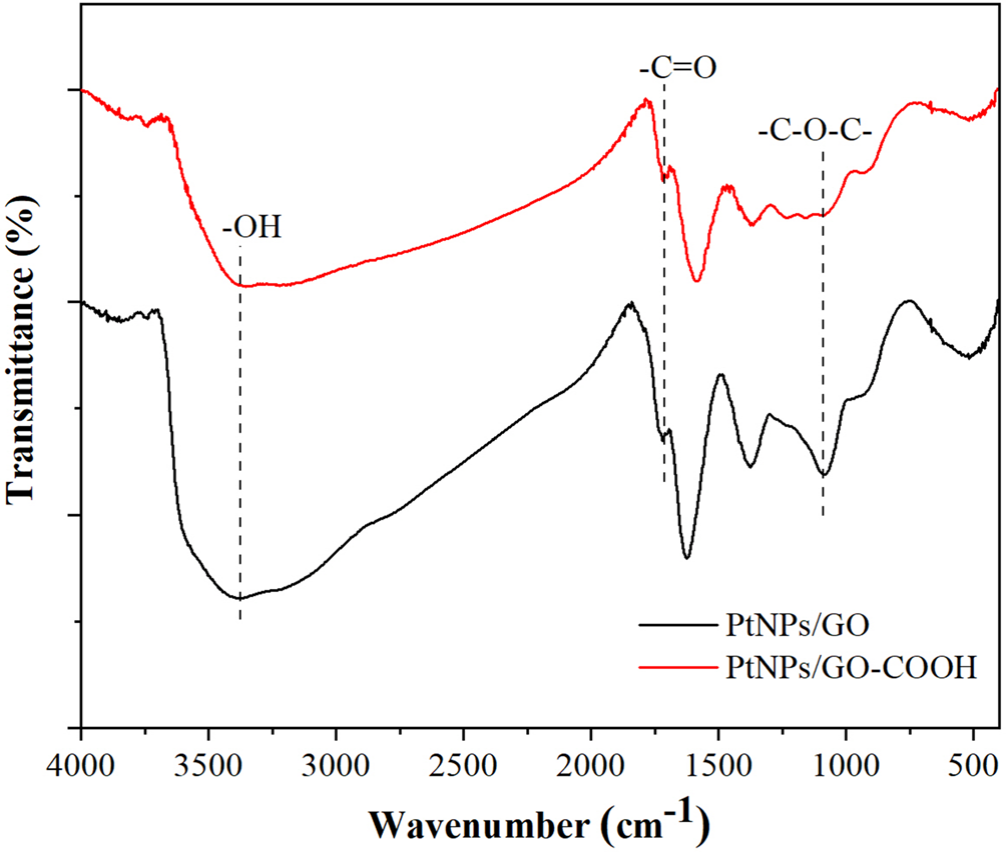
FTIR spectra of PtNPs/GO and PtNPs/GO-COOH.

The FTIR spectra were investigated to characterize the functional groups of the nanocomposites of PtNPs/GO and PtNPs/GO-COOH. The FTIR spectrum of PtNPs/GO consist of bands associating to C–O (1082 cm^−1^), C=C (1624 cm^−1^), C=O in carboxylic acid that is present along the sheet edges (1719 cm^−1^), and a broad peak between 3000 and 3500 cm^−1^ corresponding to O–H vibration, as depicted in Fig. 3. After the carboxylation of GO, the similarly spectrum was obtained with decreasing of C–O–C band, indicating that the GO-COOH was successfully synthesized. Such FTIR spectrum is a characteristic of GO-COOH. The presence of polar functional groups of the synthesized PtNPs/GO-COOH provides excellent hydrophilic property.

**Figure 4:**
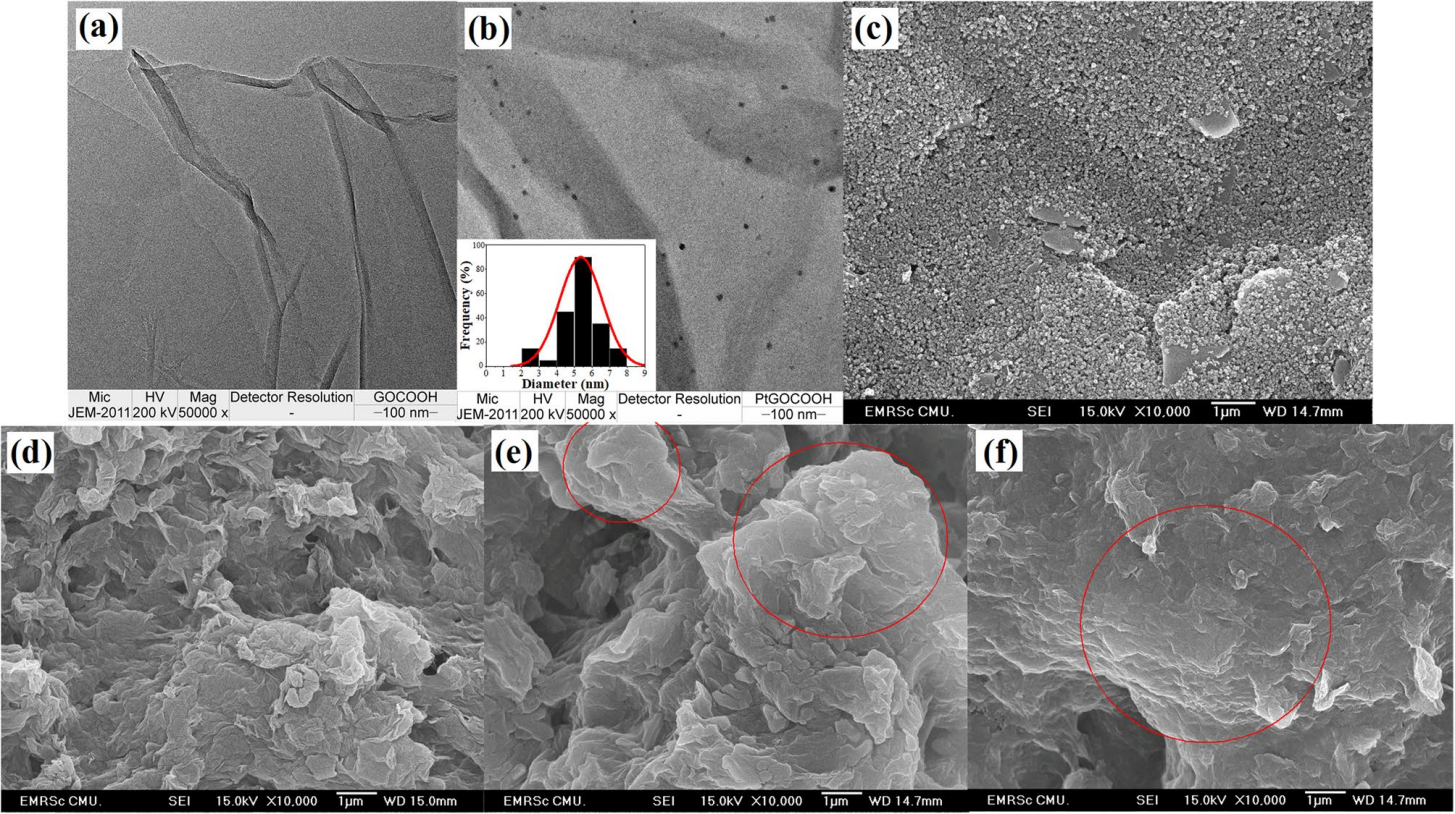
TEM images of (**a**) GO-COOH and (**b**) PtNPs/GO-COOH, and SEM images of (**c**) bared SPGE, (**d**) PtNPs/GO-COOH/SPGE, (**e**) Apt/PtNPs/GO-COOH/SPGE, and (**f**) AFP/BSA/Apt/PtNPs/GO-COOH/SPGE.

Then, the morphology of PtNPs/GO-COOH was characterized by TEM. The GO-COOH is a smooth thin sheet with a few wrinkles (Fig. 4a). After reducing platinum ions onto GO-COOH, Pt nanoparticles are well dispersed on the graphene oxide sheet with an average size of 5.6 + 1.1 nm, as shown in Fig. 4b. Furthermore, the surface of bared and the modified electrodes at different steps of the aptasensor preparation were characterized by the SEM. A reasonably uniform rough surface is displayed for a bared SPGE (Fig. 4c). After modifying with PtNPs/GO-COOH, its surface is completely covered with sheets of GO-COOH (Fig. 4d), which offer a large surface area. When the aptamer and AFP protein were immobilized onto the modified electrodes, the appearance of thicker layers (shown in the circle) were observed in Fig. 4e,f. It may be the adsorbed aptamers and complexes of AFP bound with the aptamers, which increase the surface thickness.

In addition, the immobilization of biomolecules at each step can be confirmed by considering the nitrogen element content, which is representative of biomolecular composition. When the aptasensor (BSA/Apt/PtNPs/GO-COOH/SPGE) was incubated with AFP, nitrogen's content should increase as a successful binding of the protein. The results in Fig. S1a and S1b (in supporting information) showed that the nitrogen contents before and after AFP incubation were 4.11% and 6.47%, respectively. While EDS mapping results were also in good agreement as larger spots of nitrogen were obtained, suggesting that AFP was attached to the aptasensor.

**Figure 5:**
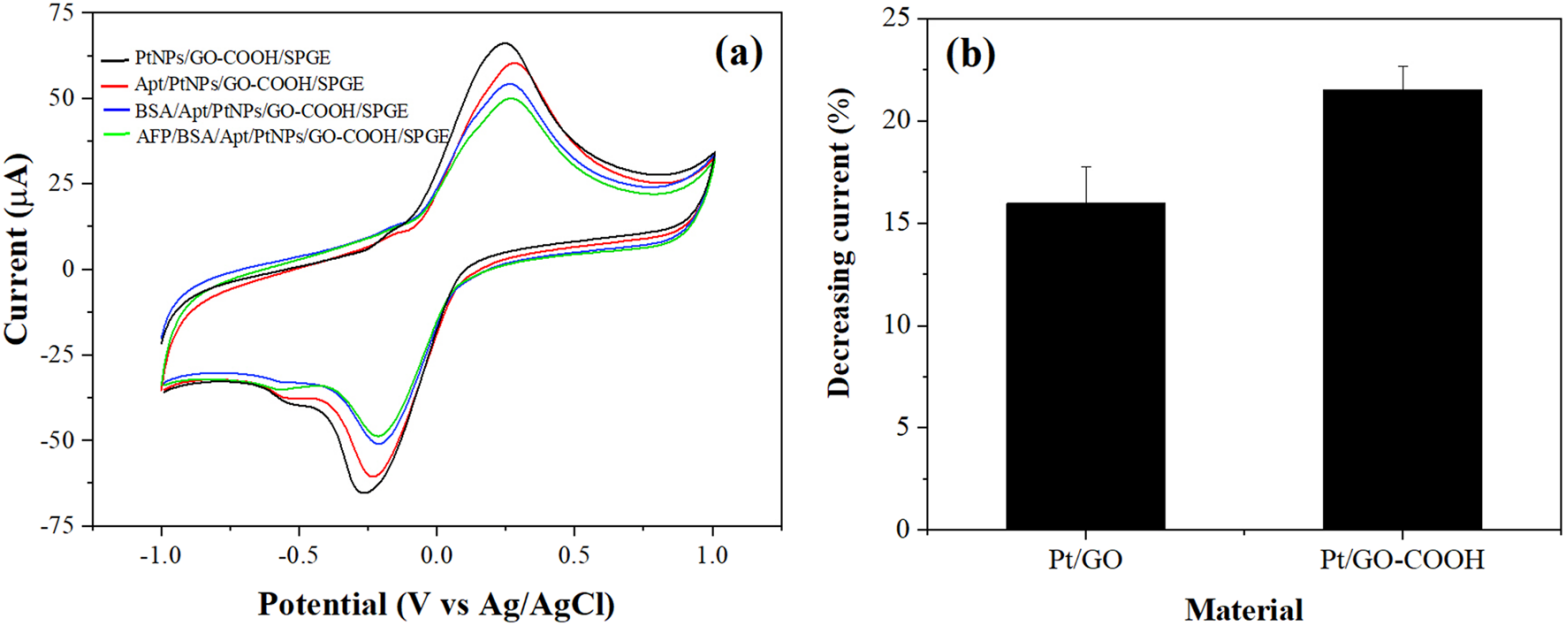
(**a**) CVs of the different modified electrodes in 10 mM PBS solution pH 7.4 containing 3 mM hydroquinone at a scan rate of 100 mV s^−1^ and (**b**) the current response of the BSA/Apt/PtNPs/GO/SPGE and the BSA/Apt/PtNPs/GO-COOH/SPGE to AFP 50 ng mL^-1^.

The cyclic voltammograms of the electrode at different immobilization steps were investigated to explore the feature of each modified electrode. Figure 5a (black line) shows anodic and cathodic peaks of hydroquinone at the PtNPs/GO-COOH/SPGE. When aptamers were immobilized, a decrease in peak currents is observed, as shown in Fig. 5a (red line), because the immobilized aptamers lower the electron transfer at the solution/electrode interface. After BSA incubation, the current further decreased, as can be observed in Fig. 5a (blue line), since the non-specific binding area was blocked. Finally, the peak currents decreased again after incubation of AFP (Fig. 5a, green line) as the complex formed by the aptamer and AFP hinders more the electron transfer of the sensing electrode. The result can indicate that AFP molecules were successfully bound with the aptamers.

In addition, the effectiveness of carboxylation on GO to load the aptamers was studied by comparing the percentage of decreasing current responses of the AFP detection (50 ng mL^−1^) from the Aptamer/PtNPs/GO/SPGE and the Aptamer/PtNPs/GO-COOH/SPGE. A result shown in Fig. 5b presents that the aptasensor utilized the PtNPs/GO-COOH provided a higher response than using PtNPs/GO. It suggests that the abundance of carboxylic groups on the GO-COOH can increase NH_2_-aptamers immobilization via the covalently binding, leading to better sensitivity to AFP detection.

### Optimization of aptamer concentration and AFP incubation time

Some parameters of the aptasensor fabrication were optimized to achieve high sensitivity for the detection of AFP protein. Firstly, the effect of the aptamer concentration during aptamer immobilization was studied. To this end, the % decreasing current of the different modified electrodes was evaluated after incubation with a 50 ng mL^−1^ AFP protein solution. The percentage of decreasing current reaches a maximum at an aptamer concentration of 7 µM as presented in Fig. S2a hereafter because the electrode surface should be saturatedly immobilized at a higher concentration. So, the aptamer concentration of 7 µM was selected as the optimum aptamer concentration.

Then, the incubation time of AFP was investigated in a range of 15 to 120 min at room temperature. The result presented in Fig. S2b implies that the current response decreases more when incubation time increases from 15 to 45 min, and reaches a maximum at 45 min. Indeed, short incubation times are not enough to completely form complexes between aptamer and AFP protein. Thus, the optimal AFP incubation time of 45 min was chosen for further experiments.

### Analytical performance of the electrochemical aptasensor

**Figure 6:**
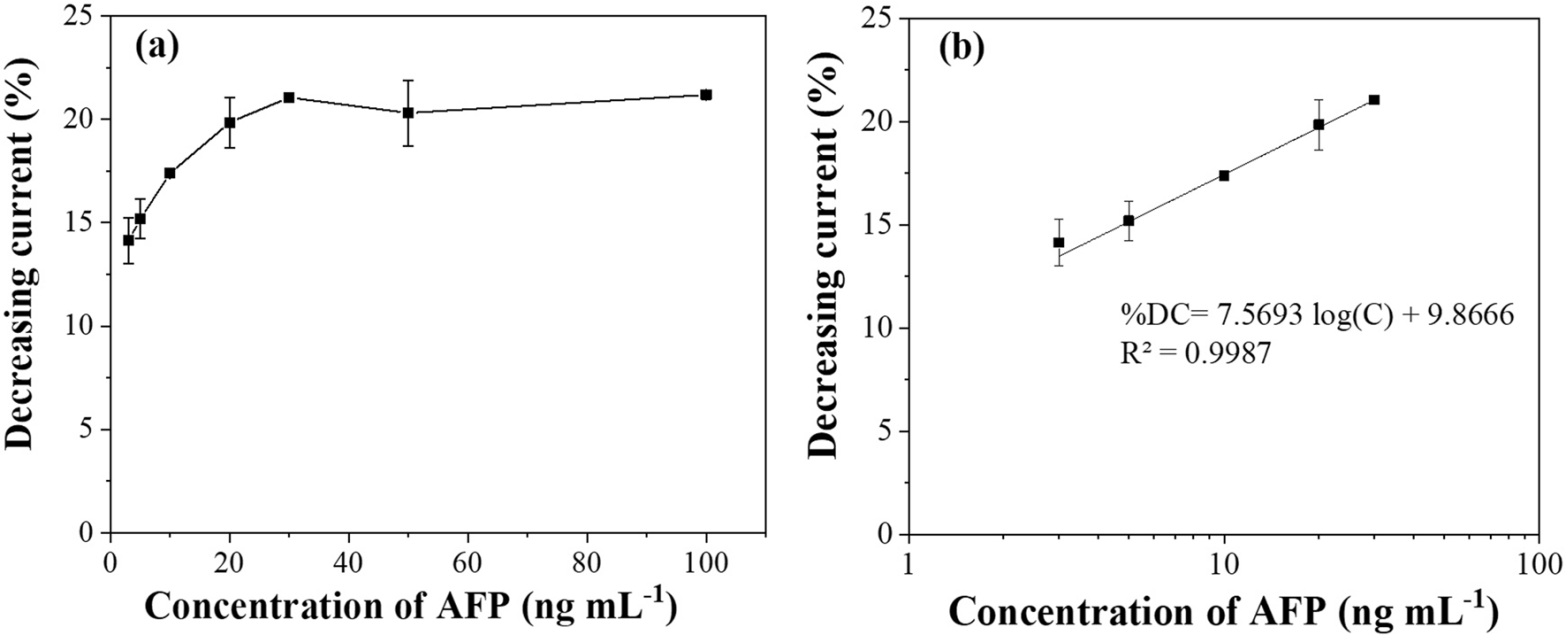
(**a**) A relationship between % decreasing current and different AFP concentrations and (**b**) the calibration graph for AFP detection.

Under the optimal conditions, the performance of the prepared BSA/Apt/PtNPs/GO-COOH/SPGE toward AFP determination was investigated by SWV. The percentages of decreasing current proportionally increase with AFP concentrations because aptamer and AFP interaction can act as an insulator and hinder the electron transfer of the electrode, inhibiting the electrochemical reaction of the redox probe. The % decreasing current (%DC) began to be stable at AFP concentration of 30 ng mL^−1^ onward, as shown in Fig. 6a, due to the saturation of AFP on the electrode surface. The calibration curve in Fig. 6b presents that % decreasing current values linearly related to the logarithm of AFP concentration from 3 to 30 ng mL^−1^ with a detection limit of 1.22 ng mL^−1^ based on a three-fold standard deviation of the intercept divided by the slope of a calibration graph.

Furthermore, a comparison of the analytical performances of various AFP electrochemical sensors using either antibodies or aptamers is summarized in Table 1. It can be seen that the proposed aptasensor is comparable to the previous electrochemical sensors although it does not offer the lowest LOD and the broadest linear range. The use of aptamer and SPGE provides advantages as above mentioned in the introduction, compared to biorecognition based on antibodies and the GCE platform. The use of SPGE modified with PtNPs/GO-COOH obviously showed lower LOD than SPCE modified with TH/RGO/AuNPs as the prominent properties of the fabricated SPGE and the synthesized nanomaterials in this work. The low LOD of 1.22 ng mL^−1^, makes it possible for the application to determine AFP in diluted serum samples. Moreover, the proposed sensor offers simple preparation and detection procedure, fast and cost-effective.

**Table 1:** Comparison of the analytical performance of different label-free electrochemical immunosensors for AFP detection.

Modified electrode	Technique	Linear range (ng mL^−1^)	LOD (ng mL^−1^)	Ref
BSA/AFP-Ab/SnO_2_/Au/RGO/GCE	DPV	0.02–50	0.01	^35^
BSA/AFP-Ab/Pd–rGO/GCE	DPV	0.01–12	0.005	^36^
BSA/AFP-Ab/AuNPs/PGNR/GCE	DPV	5–60	1.0	^37^
BSA/AFP-Apt/TH/RGO/AuNPs/SPCE	DPV	100–100,000	50	^2^
BSA/AFP-Apt/GO/GCE	CV	0.01–100	0.003	^17^
BSA/AFP-Apt/PtNPs/GO-COOH/SPGE	SWV	3–30	1.22	This work

*Ab* antibody, *Apt* aptamer, *Pd–rGO* palladium–reduced graphene oxide, *PGNR* porous graphene nanoribbon, *TH/RGO/AuNPs* thionin/reduced graphene oxide/gold nanoparticles.

### Reproducibility, selectivity, and stability

To investigate the reproducibility of the developed aptasensor, seven biosensor electrodes (BSA/Apt/PtNPs/GO-COOH/SPGEs) were prepared to detect 10 ng mL^−1^ of AFP. The relative standard deviation (RSD) of the % decreasing current of the seven electrodes was 4.36%, indicating high reproducibility of the aptasensor preparation.

After that, some biological molecules, including hepatitis B surface antigen (HBsAg), immunoglobulin G (IgG), prostate-specific antigen (PSA), and bovine serum albumin (BSA); were incubated to the BSA/Apt/PtNPs/GO-COOH/SPGE to study the selectivity of the proposed aptasensor. The percentages of decreasing current obtained from 10 ng mL^−1^ of AFP and 200 ng mL^−1^ of HBsAg, IgG, PSA, and BSA were compared as shown in Fig. S3 (in supporting information). It was found that the four electrodes incubated with other substances exhibited a low % decreasing current response comparing to the one incubated with AFP, suggesting that the fabricated sensor had good selectivity to AFP detection.

In addition, the stability of the proposed sensor was investigated by preparing several electrodes (BSA/Apt/PtNPs/GO-COOH/SPGEs) and keeping them in a refrigerator at 4 °C before taken to incubate with AFP at different period of times. The response of the aptasensor was slightly decreased as depicted in Fig. S4 (in supporting information) because some aptamers possibly self-hybridized which further provided a low efficiency of the aptamer-target AFP protein interaction. The 77.8% of its initial response after seven days can be maintained, implying fairly good stability of the aptasensor.

### Determination of AFP in real sample

Practical application of the developed aptasensor for AFP determination was demonstrated by recovery investigation of the serum sample. A human serum sample was diluted 100 folds with 10 mM PBS solution in order to avoid interference of non-specific proteins in the serum. As previously reported, the AFP level for HCC screening is about 500 ng mL^−1^, so the as-prepared biosensor can be applicable to detect AFP in the diluted serum sample. All experiments were performed in triplicate. Various concentrations of AFP were spiked into the diluted human serum samples, and the current responses were determined by SWV under the optimum conditions. As a result, the percentages of recoveries were found in the range of 97.0 to 108.9, as reported in Table 2, indicating that the proposed sensor is reliable for detecting AFP.

**Table 2:** Determination of AFP in human serum samples.

Sample	Spiked AFP concentration (ng mL^−1^)	Found AFP concentration (ng mL^−1^)	Recovery (%)	RSD (%)
1	3.0	2.91	97.0	4.88
2	10.0	10.89	108.9	2.83
3	20.0	20.85	104.2	3.74

## Conclusion

PtNPs decorated on carboxylated GO were successfully synthesized to modify on SPGE for use in a facile electrochemical aptasensor. The AFP aptamer was immobilized on the modified electrode to act as a bio-recognition molecule for the detection of AFP protein. The PtNPs/GO-COOH modified SPGE significantly enhances the current response of the hydroquinone electrochemical probe compared to bared SPGE and PtNPs/GO/SPGE. Besides, the addition of carboxylic acid groups on GO can increase the aptamer molecules immobilized on the electrode leading to provide better sensitivity of the sensor. The fabricated biosensor showed satisfying analytical performances, including simple preparation, good sensitivity (a detection limit of 1.22 ng mL^−1^), and considerable reproducibility (4.36%RSD for 7 prepared aptasensors). Owing to outstanding properties of aptamer, the AFP aptamer immobilized modified electrode offered high selectivity to AFP protein and good stability for 7 days. Furthermore, the acceptable recoveries suggested that the sensor can be applied to determine AFP in real human serum samples.

## Supplementary Information

Supplementary Information.
