# Supplementary material for: A simple label-free electrochemical sensor for sensitive detection of alpha-fetoprotein based on specific aptamer immobilized platinum nanoparticles/carboxylated-graphene oxide
Source: Sci Rep. 2021 Jul 7;11:13969. doi: 10.1038/s41598-021-93399-y (PMC8263621; doi:10.1038/s41598-021-93399-y)
Supplement: Supplementary file 1 — Supplementary Information. [file 41598_2021_93399_MOESM1_ESM.pdf]

Supplementary Information

A simple label-free electrochemical sensor for sensitive detection of alpha-fetoprotein based on specific aptamer immobilized platinum nanoparticles/carboxylated-graphene oxide

Jantima Upan[1], Napaporn Youngvises[2], Adisorn Tuantranont[3,4], Chanpen Karuwan[3,4], Philippe Banet[5], Pierre-Henri Aubert[5], Jaroon Jakmunee[1,4,6*]

[1]*Department of Chemistry, Faculty of Science, Chiang Mai University, Chiang Mai, 50200, Thailand*

[2]*Thammasat University Research Unit in Carbon Materials and Green Chemistry Innovations, Department of Chemistry, Faculty of Science and Technology, Thammasat University, Pathum Thani, 12120, Thailand*

[3]*Graphene and Printed Electronics for Dual-Use Applications Research Division, National Security and Dual-Use Technology Center, National Science and incorTechnology Development Agency, Pathumthani, 12120, Thailand*

[4]*Center of Advanced Materials of Printed Electronics and Sensors, Materials Science Research Center, Faculty of Science, Chiang Mai University, Chiang Mai 50200, Thailand*

[5]*CY Cergy Paris Université, LPPI, F-95000, Cergy-Pontoise, France*

[6]*Center of Excellence for Innovation in Chemistry, and Center of Chemistry for Development of Health Promoting Products from Northern Resources, Faculty of Science, Chiang Mai University, Chiang Mai, 50200, Thailand*

* e-mail: jaroon.jakmunee@cmu.ac.th, jakmunee@gmail.com

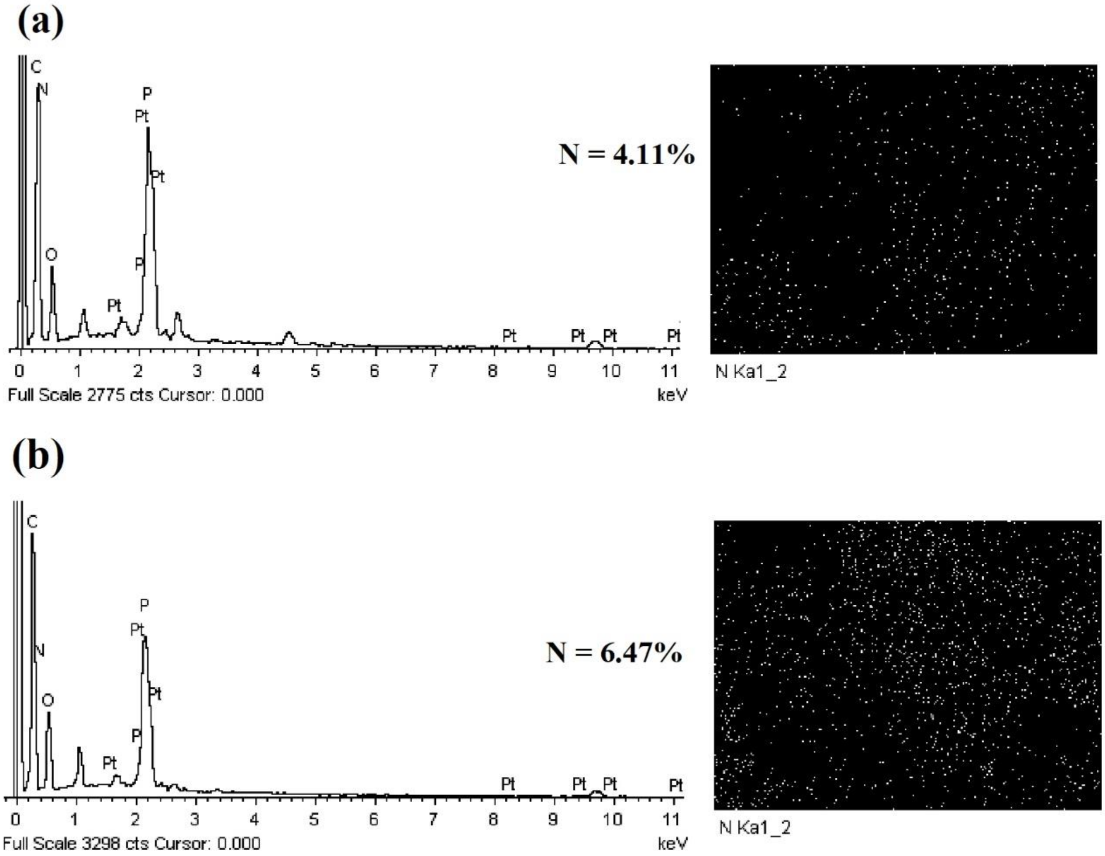

Figure S1. EDS spectra and mapping of (a) BSA/Apt/PtNPs/GO-COOH/SPGE and (b) AFP/BSA/Apt/PtNPs/GO-COOH/SPGE.

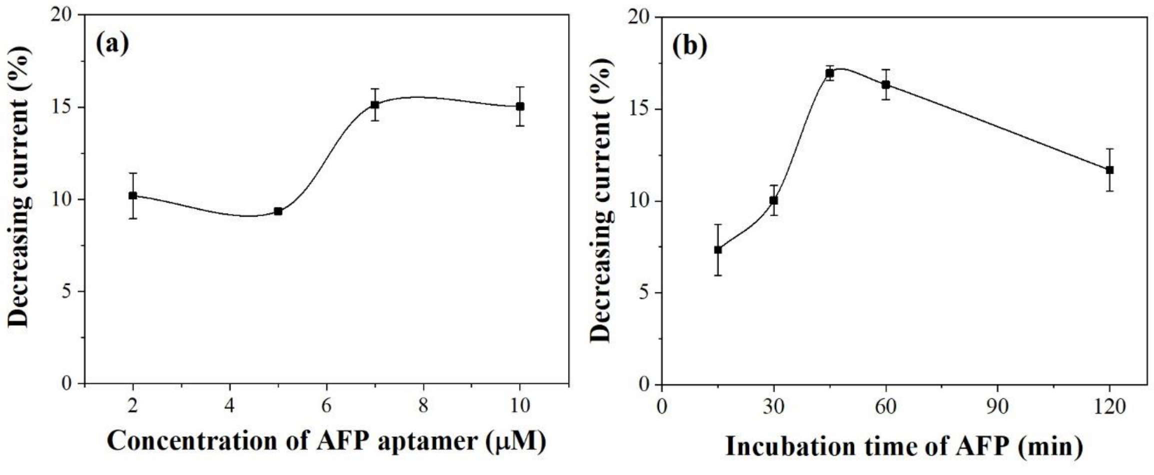

Figure S2. (a) Effect of aptamer concentration and (b) incubation time of AFP on the current response of aptasensor.

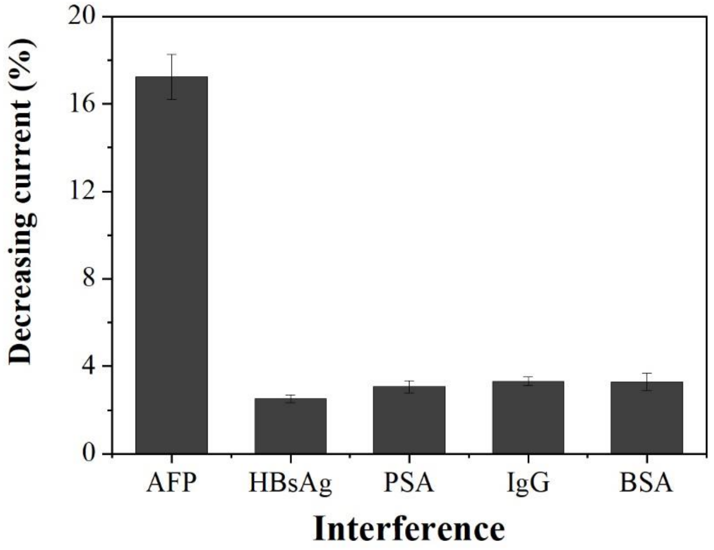

Figure S3. The response of the aptasensor to 10 ng mL^{-1} of AFP, and 200 ng mL^{-1} of HBsAg, PSA, IgG, and BSA.

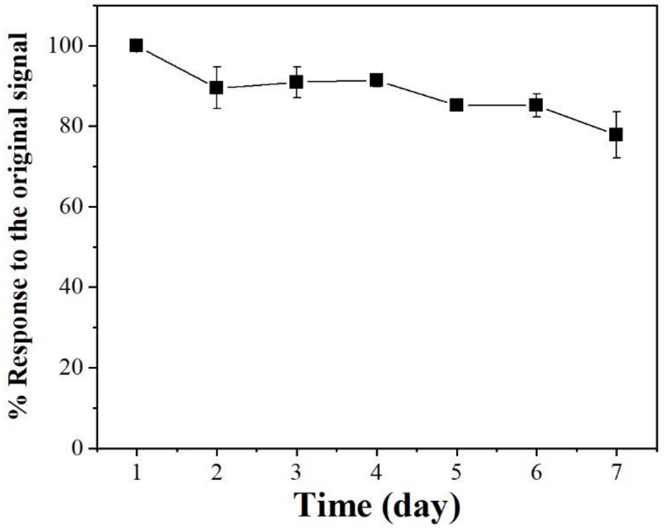

Figure S4. Stability of the AFP aptasensor.
